# Superior Vena Cava Syndrome as the Initial Manifestation of a Thoracic Aortic Aneurysm: A Case Report

**DOI:** 10.7759/cureus.96380

**Published:** 2025-11-08

**Authors:** Anass M'Ghari, Omar Nafii, Mohamed Sarsari, Houda Bachri, Jamila Zarzur

**Affiliations:** 1 Cardiology Department, Ibn Sina University Hospital, Mohammed V University, Rabat, MAR

**Keywords:** ascending aortic aneurysm, bicuspid aortic valve, case report, chronic inflammation, superior vena cava syndrome, thoracic aortic aneurysm, venous thrombosis

## Abstract

Superior vena cava syndrome (SVCS) is caused by impaired venous return from the head, neck, and upper extremities, most commonly due to mediastinal malignancies. Benign causes such as thoracic aortic aneurysm (TAA) are rare, and presentation as SVCS is exceptionally uncommon.

We report the case of a 39-year-old chronic smoker presenting with progressive facial, cervical, and upper-extremity swelling and morning orthopnea. Examination revealed prominent chest-wall collateral veins suggestive of SVCS. CT angiography demonstrated a 5.5-cm ascending aortic aneurysm compressing the superior vena cava, with associated venous thrombosis and collateral circulation. A comprehensive work-up excluded infectious, autoimmune, and neoplastic causes, and positron emission tomography (PET) imaging confirmed inflammatory activity around the aneurysm without evidence of malignancy.

The patient received anticoagulation and beta-blockade followed by elective surgical repair with restoration of caval flow. Postoperative recovery was uneventful, and histopathology confirmed nonspecific chronic inflammation of the aortic wall.

This case highlights that TAA, even when modest in size, may rarely cause SVCS and underscores the importance of considering vascular causes in the differential diagnosis. It further emphasizes the value of timely multidisciplinary evaluation in achieving favorable outcomes.

## Introduction

Superior vena cava syndrome (SVCS) is characterized by swelling of the face, neck, and upper extremities and dyspnea. These symptoms result from obstruction of venous return through the superior vena cava (SVC), leading to collateral venous circulation visible on examination [1]. SVCS affects approximately 15,000 patients annually in the United States and is most commonly associated with mediastinal malignancies, particularly lung cancer and lymphomas [1,2]. Although malignant etiologies predominate, benign causes are increasingly recognized, in part due to the expanding use of intravascular devices [3].

Thoracic aortic aneurysms (TAA) are defined by ≥50% dilation of the thoracic aorta and typically remain clinically silent until complications arise [4]. Current surgical thresholds are based on balancing the risk of rupture or dissection against operative risk, and many aneurysms are monitored until reaching guideline-recommended size criteria [5].

TAA presenting as SVCS is exceedingly rare, and most reported cases involve very large or dissecting aneurysms. The novelty of this case lies in the presentation of SVCS caused by a comparatively modest ascending TAA (5.5 cm) without dissection. Recognizing this uncommon mechanism is essential, as delayed diagnosis may lead to catastrophic consequences. This report adds to the limited literature by demonstrating that even non-complicated, moderate-sized aneurysms can precipitate SVCS and should remain in the differential diagnosis when vascular compression is suspected.

## Case presentation

A 39-year-old male smoker presented with progressive swelling of the face, neck, chest, and upper extremities, worse in the mornings and with activity, accompanied by transient morning orthopnea. No significant past medical history or systemic disease was reported.

Examination revealed facial, neck, and upper-extremity swelling with prominent chest-wall collateral veins, consistent with SVCS.

Chest X-ray showed mediastinal widening and a prominent aortic knob. C-reactive protein (CRP) was elevated at 40 mg/L; other labs were unremarkable.

**Figure 1:**
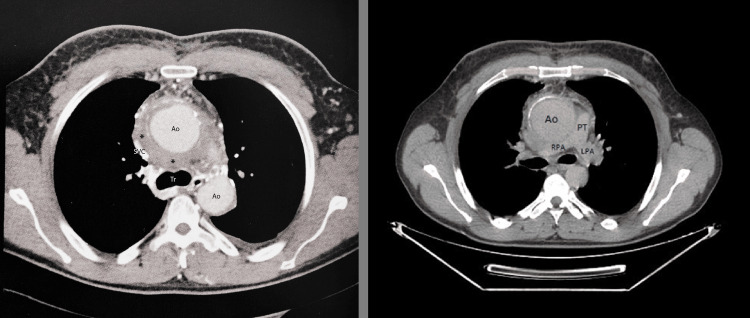
Angiogram (left) and non-contrast CT (right) showing aneurysmal dilation of the ascending aorta with periaortic infiltration (*) causing lamination of the SVC Ao: Thoracic Aorta; SVC: Superior Vena Cava; Tr: Tracheal Carina; PT: Pulmonary trunk; RPA: Right Pulmonary Artery; LPA: Left Pulmonary Artery

**Figure 2:**
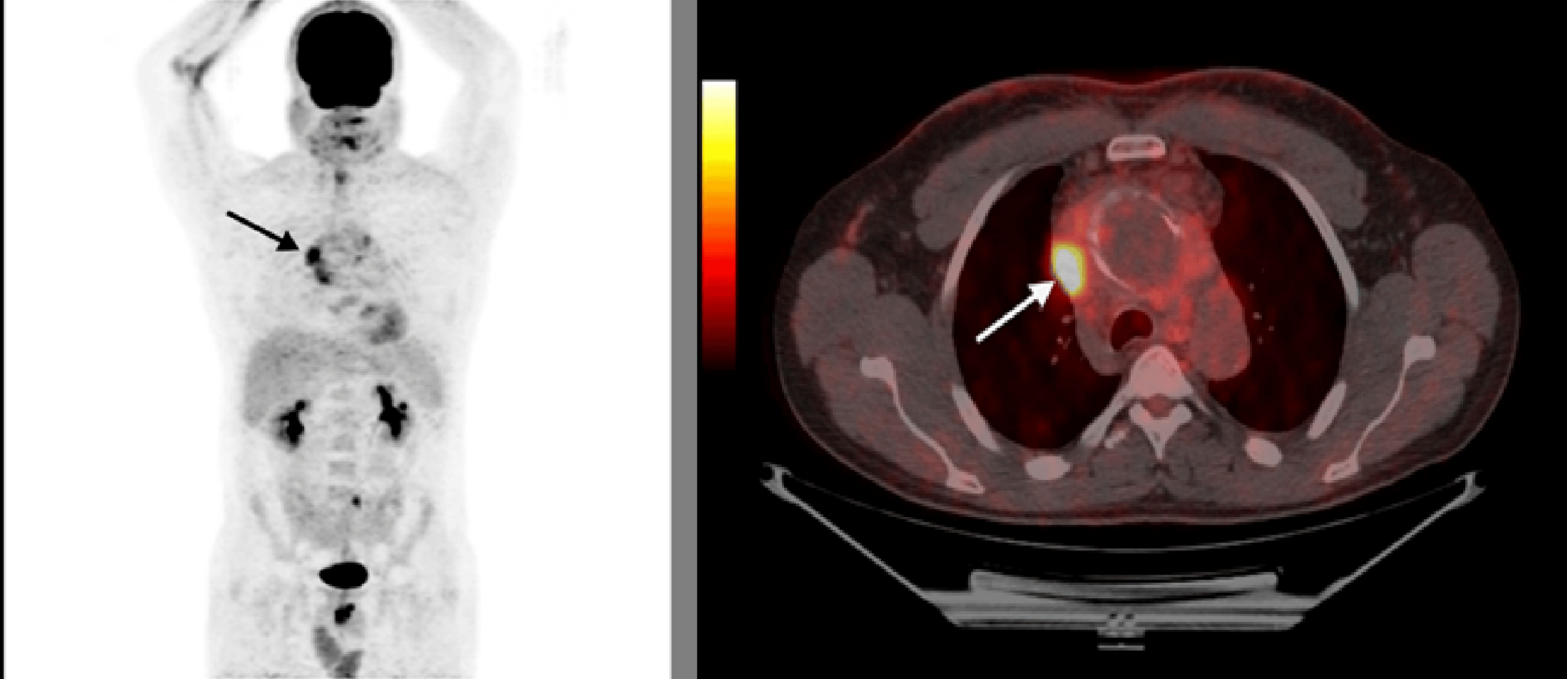
MIP (left) and PET/CT (right) images demonstrate marked right mediastinal hypermetabolism (arrows) related to SVC thrombosis and inflammation MIP: Maximal inspiratory pressure, PET/CT: Positron emission tomography-computed tomography, SVC: Superior vena cava

CT angiography (Figure 1) revealed a 55 mm aneurysm of the ascending aorta, associated with SVC thrombosis, collateral venous circulation, and a small left pleural effusion. A second review of the imaging noted enlargement of cervical and mediastinal lymph nodes, with ultrasound and subsequent biopsy confirming normal lymphoid tissue and an inflammatory process, without evidence of neoplastic or granulomatous disease. Complimentary transthoracic echocardiography (TTE) (Figure 2) showed marked dilation of the ascending aorta with suspicion of a type 0 anteroposterior aortic bicuspid valve with no significant aortic valve dysfunction.

After 18 days of therapeutic low-molecular-weight heparin (LMWH), a follow-up non-contrast CT of the chest, abdomen, and pelvis (CT NCAP) was performed to evaluate for malignancy. Although no sign of the latter was identified, the scan confirmed the fusiform ascending aortic aneurysm measuring 58 mm, extending from the aortic root to the origin of the left common carotid artery. The aneurysm demonstrated hemi-circumferential atheromatous calcification and mild periaortic infiltration, resulting in a mass effect on the flattened superior vena cava, with no thrombus identified within the vessel.

Positron emission tomography (PET) scan (Figure 2) demonstrated pathological hypermetabolism in the right mediastinum, likely related to SVC thrombosis, with two small hypermetabolic foci in the peri-aneurysmal region, probably inflammatory in origin. No findings suggestive of malignancy were identified elsewhere.

Etiological workup excluded infectious, autoimmune, and neoplastic causes. Plasma protein electrophoresis indicated chronic inflammation, consistent with persistently elevated CRP levels.

In summary, this case describes a 39-year-old male with SVCS caused by a moderately sized, non-complicated ascending aortic aneurysm compressing the SVC with associated thrombosis in the setting of chronic inflammation.

**Figure 3:**
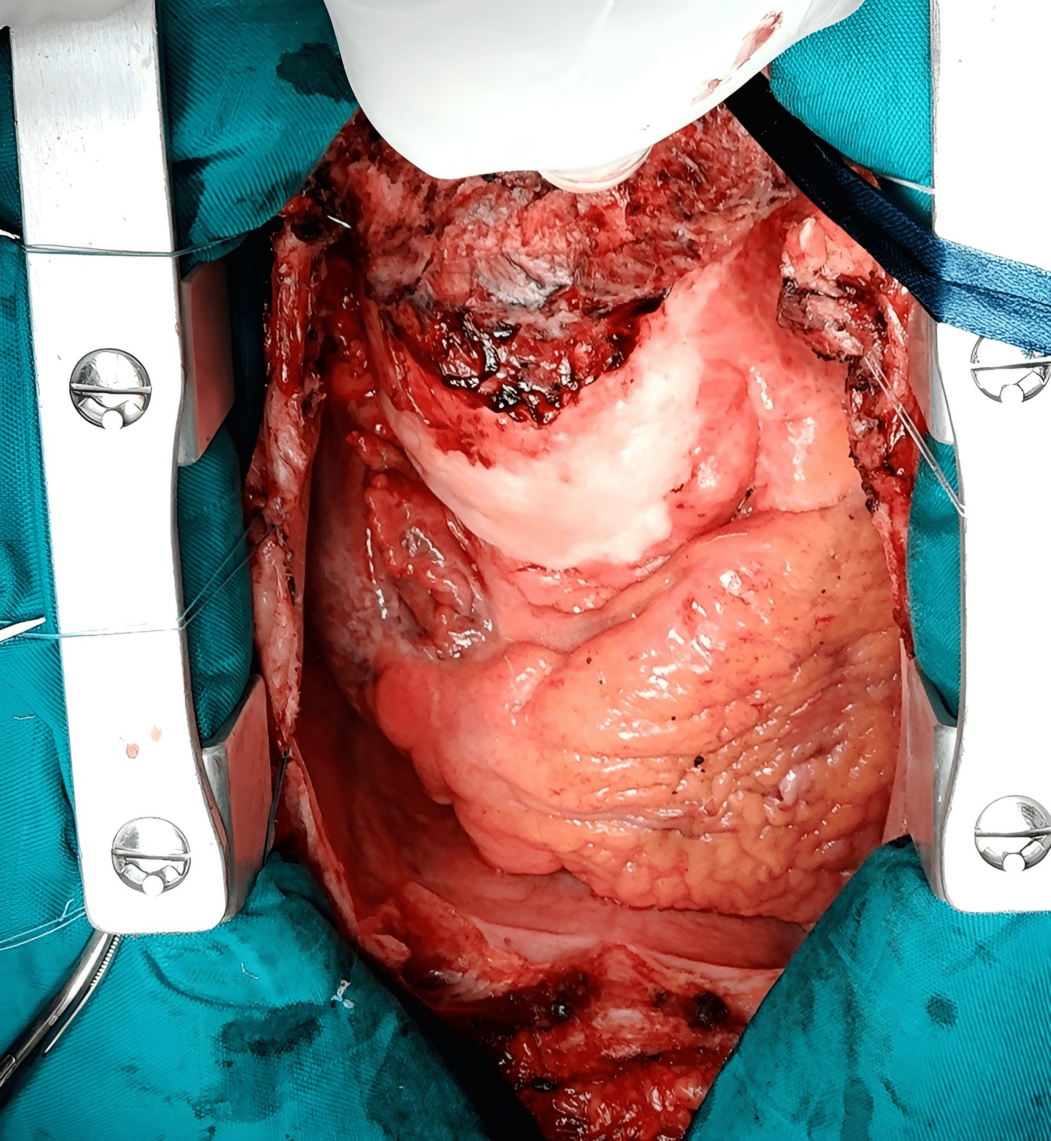
Intraoperative view showing marked calcification of an enlarged aortic root with infiltration of the ascending aorta

The patient was initiated on therapeutic LMWH and beta-blockers. Following multidisciplinary consultation with the Heart Team, elective repair of the aneurysm was performed at the Cardiology Center using the Tirone David procedure under cardiopulmonary bypass (CPB). Intraoperatively, the aortic valve was tricuspid, with marked calcification of the aortic root, and infiltration and thickening of the ascending aorta were observed. (Figure 3). The ascending aorta was clamped and replaced with a Dacron graft, and the superior vena cava (SVC) was cleared of residual thrombus. CPB was successfully weaned without postoperative complications, and the patient made a full recovery. Histopathology of the excised aortic wall demonstrated nonspecific chronic inflammation.

## Discussion

SVCS is currently primarily caused by compression and invasion of the SVC by adjacent mediastinal malignancies, chiefly small cell bronchogenic carcinomas and lymphomas [2]. First described by William Hunter in 1757 in the context of syphilitic aortic aneurysm, SVCS was historically driven by benign etiologies that have since become rare with advances in antibiotic treatment and overall longevity [1].

SVC thrombosis is an increasingly common cause of benign SVCS. This is mostly associated with the use of indwelling central venous catheters and pacemaker leads, although spontaneous clot formation may occur due to hypercoagulable states, such as in the presence of malignancy or chronic inflammation, which warranted a full etiological assessment in our patient [6,7].

A review of previously reported cases of SVCS secondary to thoracic aortic aneurysm (Table 1) demonstrates that nearly all involved either mainly degenerative giant aneurysms (>8-10 cm) or complicated aortic pathology, such as dissections or pseudoaneurysms in the postoperative setting (after aortic valve replacement or aortic aneurysm repair). Outcomes varied, with mortality occurring primarily as a result of misdiagnosis or post-mortem presentation.

**Table 1:** Summary of previously reported cases of superior vena cava syndrome associated with thoracic aortic aneurysm

Cases	Age (yrs), sex	Aneurysm diameter (mm)	Etiology	Outcome	Treatment
Philips et al. (1981) [8]	65, male	140	Syphilitic aortitis (on autopsy)	Died	Radiation therapy (misdiagnosed as malignancy)
Sekine et al. (2015) [9]	52, male	79	Likely syphilitic (positive serology)	Recovered	Surgery (arch replacement + elephant trunk)
Moutakiallah et al. (2014) [10]	72, male	110	Degenerative (idiopathic)	Recovered	Surgery (modified Bentall)
Bicer et al. (2020) [11]	77, female	140	Degenerative (idiopathic)	Recovered	Surgery (ascending aortic replacement)
Stajnic et al. (2001) [12]	60, male	92	Dissecting aneurysm (post-aortic valve replacement)	Died	— (presentation to autopsy)
Dedeilias et al. (2010) [13]	79, male	130 (pseudoaneurysm diameter)	Pseudoaneurysm (distal graft anastomosis)	Recovered	Reoperation (repaired leaks)
Morii et al. (2011) [14]	64, male	72 (pseudoaneurysm diameter)	Pseudoaneurysm (penetrating atherosclerotic ulcer)	Recovered	Surgery (resection and graft)
Dayan et al. (2008) [15]	82, male	110	Degenerative (idiopathic)	Unknown (not reported)	Not reported
Vydt et al. (2005) [16]	56, male	100 (pseudoaneurysm diameter)	Pseudoaneurysm (post-AVR)	Recovered	Surgery (repair of pseudoaneurysm)

In contrast, our case details a significantly smaller non-complicated thoracic aneurysm measuring only 5.5 cm that caused SVCS. We believe this was due both to the mechanical mass effect of the aneurysm and the thick peri-aortic infiltration surrounding the vessel, which increased the overall compressing mass. SVC thrombosis may have subsequently occurred via lamination of the vessel and hypercoagulability induced by chronic systemic inflammation, rather than intraluminal invasion as is common with malignant processes.

As per the PET scan findings in this case, and although malignant lesions tend to show higher FDG uptake on average than inflammatory processes, there is substantial overlap between the two, meaning it alone is insufficient for definitive differentiation [17]. Malignancy was therefore ruled out postoperatively after a proper histopathological exam.

Thoracic aortic aneurysms carry a risk of rupture and dissection, and timely surgical repair is crucial to prevent these complications. According to the 2022 American College of Cardiology/American Heart Association (ACC/AHA) guidelines, repair is generally recommended for ascending aortic aneurysms measuring ≥5.5 cm in patients without additional risk factors, with lower thresholds considered in high-risk situations such as connective tissue disorders, bicuspid aortic valves with risk features, rapid growth (>0.3-0.5 cm/year), or family history of aortic dissection. Symptomatic aneurysms or those causing compressive complications, regardless of size, also warrant intervention. Decisions should be individualized, taking into account patient-specific characteristics (age, comorbidities, body size) as well as aneurysm morphology and growth dynamics, and should ideally involve a multidisciplinary aortic team [18].

## Conclusions

Thoracic aortic aneurysms develop silently and remain, in most cases, asymptomatic, often being revealed only by life-threatening complications, which underscores the need for improved strategies for earlier diagnosis. In this report, we present a non-complicated thoracic aortic aneurysm (≈5.5 cm) that was identified in a timely manner thanks to an uncommon clinical finding: superior vena cava syndrome. This case illustrates that even a modest aneurysm size can cause compressive complications and that prompt multidisciplinary evaluation is essential for optimal management. We hope this report will raise awareness of these considerations and emphasize that successful care for such complex cases is best achieved through an interdisciplinary approach, in accordance with current guidelines.
